# Diagnostic approach to heart failure in Türkiye

**DOI:** 10.55730/1300-0144.5932

**Published:** 2024-05-07

**Authors:** Dilek URAL, Lale Dinç ASARCIKLI, İnci Tuğçe ÇÖLLÜOĞLU, Anıl ŞAHİN, Yüksel ÇAVUŞOĞLU, Mehmet Birhan YILMAZ, Sanem NALBANTGİL, Naim ATA, Mustafa Mahir ÜLGÜ, Şuayip BİRİNCİ, Selda MURAT, Emre DEMİR, Emine Arzu KANIK, Ahmet ÇELİK

**Affiliations:** 1 Department of Cardiology, Faculty of Medicine, Koç University, İstanbul, Turkiye; 2 Department of Cardiology, Faculty of Medicine, Health Sciences University, İstanbul, Turkiye; 3 Department of Cardiology, Faculty of Medicine, Karabük University, Karabük, Turkiye; 4 Department of Cardiology, Faculty of Medicine, Sivas Cumhuriyet University, Sivas, Turkiye; 5 Department of Cardiology, Faculty of Medicine, Eskişehir Osmangazi University, Eskişehir, Turkiye; 6 Department of Cardiology, Faculty of Medicine, Dokuz Eylül University, İzmir, Turkiye; 7 Department of Cardiology, Faculty of Medicine, Ege University, İzmir, Turkiye; 8 General Directorate of the Health Information Systems, Ministry of Health, Ankara, Turkiye; 9 Deputy Health Minister, Ministry of Health, Ankara, Turkiye; 10 Department of Biostatistics, Faculty of Medicine, Mersin University, Mersin, Turkiye; 11 Department of Cardiology, Faculty of Medicine, Mersin University, Mersin, Turkiye

**Keywords:** Heart failure, diagnostic tools, natriuretic peptides, nationwide study

## Abstract

**Background/aim:**

Final diagnosis of heart failure (HF) relies on a combination clinical findings, laboratory and imaging tests. The aim of this study was to review the diagnostic approach to HF in Türkiye.

**Materials and methods:**

This study is a subanalysis of the nationwide TRends-HF study, based on anonymized data from National Electronic Database between January 1, 2016, and December 31, 2022. Variables including date of birth, sex, socioeconomic development index, place of initial HF diagnosis, comorbidities, investigations, and diagnostic procedures were reported. Laboratory variables, including complete blood count, natriuretic peptides (NP), estimated glomerular filtration rate, uric acid, electrolytes, albumin, lipid profile, ferritin and hemoglobin A1c levels, and other imaging techniques (coronary angiogram [CAG], transthoracic echocardiography [TTE], chest X-ray [CXR], etc.) during the initial diagnosis and/or follow-up of HF patients, were obtained from the National Electronic Database. The diagnostic test usage rates were analyzed according to years, geographical regions, and socioeconomic regions of Türkiye.

**Results:**

The study population consisted of 2,722,151 HF patients (51.7% female, mean age 68.33 ± 14.01 years). All HF patients had at least one electrocardiogram and one TTE examination, and all underwent routine biochemical tests at least once during the follow-up period. CXR utilization rate was 93.7%, while CAG utilization rate was 17.9%. Coronary computed tomographic angiography and cardiac magnetic resonance imaging were performed in only 1.8% and 0.3% of patients, respectively. Among all Turkish HF patients, 16.3% had at least one NP measurement. The highest rate of NP use was observed in the Central Anatolia Region (21.0%), while the lowest rate was in the Aegean Region (11.7%). NP measurement during HF diagnosis revealed a rising trend over time (12.3% in 2016 vs. 26.3% in 2021).

**Conclusion:**

The widespread use of TTE at the beginning of the diagnosis and during follow-up is important for providing quality care to HF patients in Türkiye. However, detailed laboratory tests and advanced imaging methods are not utilized sufficiently, which could lead to issues in patient management.

## 1. Introduction

Diagnosis of heart failure (HF) requires demonstration of structural and functional cardiac abnormalities, corroborated by elevated natriuretic peptide levels or objective evidence of cardiogenic pulmonary or systemic congestion [1]. Making a definite diagnosis can sometimes be challenging because the symptoms and signs associated with HF often overlap with other disorders such as chronic obstructive pulmonary disease, kidney disease, anemia, and other related disorders. There is no single diagnostic test for HF; the final diagnosis relies on clinical findings combined with the results of laboratory and imaging tests.

Screening for the aetiological factors and comorbidities plays a crucial role in managing and improving the outcomes of HF patients. The aging population leads to an increase in the incidence and prevalence of primary HF etiologies, including hypertension and ischemic heart disease, as well as commonly occurring comorbidities, such as diabetes and chronic kidney disease [2–4]. Therefore, current guidelines on the diagnosis and management of HF recommend routine testing for significant associated conditions and comorbidities that may affect the treatment and clinical outcomes of patients [5, 6].

In ESC 2021 guidelines, routine assessments recommended for all HF patients include electrocardiogram (ECG), measurement of natriuretic peptides (NPs), full blood count, urea and electrolytes, thyroid function, fasting glucose and HbA1c, lipids, and iron status (transferrin saturation and ferritin) [5]. AHA/ACC/HFSA 2022 guideline recommends some additional tests, such as urine analysis and liver function tests, among other routine laboratory assessments [6]. Both guidelines underline the importance of periodical screening for iron deficiency with the measurement of ferritin and transferrin saturation levels [5,6]. Chest X-ray and echocardiography are the two routine imaging techniques. Other imaging tests are not mandatory and utilized only for specific purposes, such as determination of the underlying aetiology or severity of HF. These complimentary tests include stress echocardiography, single-photon emission CT (SPECT), computed tomography coronary angiography (CTCA), cardiac magnetic resonance (CMR) imaging, coronary angiography, right heart catheterization (RHC), and endomyocardial biopsy (EMB). Cardiopulmonary exercise testing (CPET) is recommended for advanced HF patients during evaluation for heart transplantation or mechanical circulatory support.

Appropriate use of diagnostic tests is the key to increasing the quality of care in HF. The aims of this study were to evaluate the diagnostic approach to HF in Türkiye, with a focus on the measurement of natriuretic peptides (NP) according to sex, age, and comorbidities of the patients, and to examine possible associations with the geographical area and socioeconomical status.

## 2. Materials and methods

This is a subanalysis of the nationwide TRends-HF study, based on anonymized data from the Turkish Ministry of Health’s National Electronic Database between January 1, 2016, and December 31, 2022. A detailed description of the study protocol has been published elsewhere [7]. The study protocol was approved by the Republic of Türkiye Ministry of Health (approval number: 95741342-020) and conforms to the Declaration of Helsinki. STROBE guidelines for cohort studies and a checklist were used in the preparation of this report [8].

The Republic of Türkiye Ministry of Health has governed the national electronic database, which merges all other databases, including diagnoses, laboratory results, imaging tests, hospitalizations, outpatient and emergency visits. Specific procedures and tests were recorded based on the Health Implementation Declaration codes. Variables including date of birth, sex, socioeconomic development index (SEDI), place of initial HF diagnosis, comorbidities, investigations, and diagnostic procedures were reported. Laboratory variables, including complete blood count, natriuretic peptides (BNP or NT-proBNP), estimated glomerular filtration rate (eGFR, based on chronic kidney disease [CKD]-EPI) [9], uric acid, electrolytes, albumin, triglycerides, LDL-cholesterol, ferritin, and hemoglobin A1c levels, were obtained from the National Electronic Database.

Socioeconomic region rankings were obtained from a 52-variable model in the socioeconomic status (SES)-2017 research report of the Republic of Türkiye Ministry of Industry and Technology. Provinces were classified according to their level of socioeconomic development (Supplementary Table S), which was divided into six categories (from the most affluent group [SES-1] to the most deprived group [SES-6])^1^.

Cross tables were created for categorical variables, with values expressed as the numbers of cases and percentages (%). Age and laboratory parameters at the time of the index HF diagnosis were described using the mean and standard deviation, as well as the median and interquartile range (25th–75th percentile). SPSS 25.0 software (IBM Corporation, Armonk, NY, USA) and E-PICOS AI (MedicReS, New York, NY, USA) were used for statistical analyses.

## 3. Results

The study population consisted of 2,722,151 HF patients (51.7% female, mean age 68.33 ± 14.01 years). The majority (72.2%) of HF cases were diagnosed in public health facilities affiliated with the Ministry of Health. Approximately 21% of the patients were diagnosed in private hospitals, 6% in state university hospitals, and 1% in private and foundation university hospitals.

All HF patients underwent at least one electrocardiogram and one transthoracic echocardiography (TTE) examination at some point during the follow-up period. Routine biochemical tests, including complete blood count, urea, creatinine, electrolytes, were also measured at least once in every patient. The number of patients who underwent other laboratory tests and their results are presented in Table 1.

Among all Turkish HF patients, 16.3% had at least one NP measurement. The rate of NP usage was similar for both sexes (16.9% in males vs. 15.7% in females) and did not change with increasing patient age (Table 2). The highest NP levels were observed in patients aged 75 years and older. The median BNP value was 1475 pg/mL and NT-proBNP level was 2150 pg/mL in this age group.

The type of comorbidity had a significant impact on the use of NPs (Table 3). NPs were measured more frequently in patients with obesity and CKD compared to those with diabetes or chronic obstructive pulmonary diseases. As expected, NP levels tended to increase in parallel with the decline in kidney function (Table 4).

**Figure 1:**
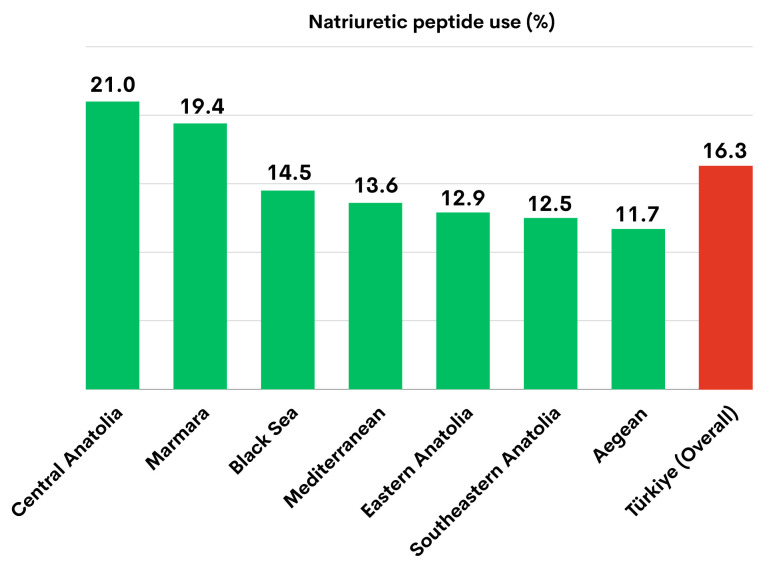
The natriuretic peptide usage rate across seven geographical regions and in Türkiye.

The analysis of NP usage rates by geographical region revealed that the Central Anatolia Region had the highest rate (21.0%), while the Aegean Region had the lowest rate (11.7%) (Figure 1).

**Figure 2:**
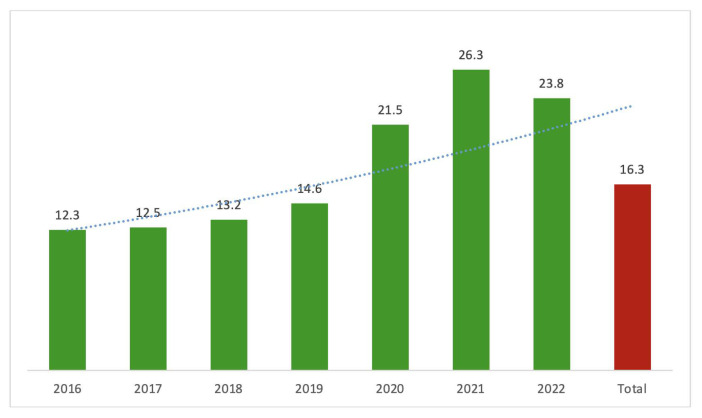
Trend of natriuretic peptide usage from 2016 to 2022 in Türkiye.

The analysis of NP measurement rates during HF diagnosis by year revealed an increasing trend in NP utilization over time, from 12.3% in 2016 to 26.3% in 2021 (Figure 2).

Among patients diagnosed with HF, imaging methods other than electrocardiogram and transthoracic echocardiography (TTE) included posteroanterior chest radiography in 93.7% of cases, and conventional coronary angiography in 17.9% of cases. Conventional coronary angiography was more frequently performed in male patients compared to female patients (22.4% vs. 13.7%, respectively).

Among the other diagnostic tests, CTCA was performed in only 1.8% of patients, CMR imaging in 0.3%, EMB in 354 (0.01%) HF patients. The distribution of imaging modalities and invasive procedures used in the diagnosis and during follow-up of HF, stratified by sex, and a comparison with EuroHeart Failure Survey in 2003 are presented in Table 5.

The socioeconomic status of the province significantly influenced the use of laboratory and imaging tests (Table 6). NP measurement was less frequently performed in the most deprived cities (SES-6). Paradoxically, however, patients in the SES-6 region managed underwent imaging tests such as CTCA and CAG more frequently than those in the most affluent group (SES-1).

## 4. Discussion

The utility of the two crucial diagnostic tests for HF demonstrated significant differences in Türkiye. While TTE, the primary imaging test, was available to every HF patient, measurement of NPs, the crucial diagnostic laboratory test, was less frequently conducted across all patient groups. Complete blood count, glucose, kidney function tests, and electrolytes were assessed in every patient either at the initial diagnosis or during follow-up; however, other routine tests, including thyroid function, HbA1c, lipids and iron status (TSAT and ferritin) were less frequently performed. Among the imaging tests other than TTE, only CAG was used with greater frequency than the others; however, CTCA and CMR were performed in only 1.8% and 0.3% of the entire HF population, respectively.

TTE and ECG are easily accessible methods in Türkiye and can be applied to all patients admitted to the cardiology clinics. TTE is widely available in most healthcare facilities and considered a ‘routine’ component of cardiovascular examination. Additionally, it is relatively inexpensive and fully covered by the national insurance system.

On the other hand, NPs, a crucial diagnostic instrument for HF, were measured significantly less frequently than recommended. Despite an increasing trend in NP measurement over the years, it was performed in only 9.6% of the patients in the SES-6 region and only slightly higher (16.3%) in the entire country. The primary factors contributing to the low NP measurement rates may include reimbursement issues related to the test, as well as the knowledge and attitudes of physicians. In Türkiye, NPs are reimbursed only when ordered by specialist physicians in internal medicine, chest diseases, cardiology, pediatrics, cardiovascular surgery or thoracic surgery. HF is most commonly diagnosed in secondary and tertiary public healthcare facilities where the mentioned specialists are in charge. However, a substantial number of patients are diagnosed with HF in primary public health care facilities. Given that a definitive diagnosis of HF relies on elevated NP levels corroborated by echocardiographic findings, expanding NP measurement services to all healthcare facilities, especially to primary care facilities, could be a valuable strategy to enhance the quality of HF care.

Providing healthcare professionals with additional information on the utility of NP measurement can enhance their awareness and perceptions on the test’s value. NP measurement is cost-effective, not dependent on personnel, has a high negative predictive value, and is frequently reproducible [10]. Additionally, it can be utilized for screening HF, particularly in people with chronic conditions such as diabetes, atherosclerotic cardiovascular disease, chronic obstructive pulmonary disease and patients with a history of cardiotoxic drug use due to oncological diseases [11–13]. In the absence of reduced EF, measuring NPs is a practical and cost-effective method for diagnosing HF with preserved EF. Secular trends over the past two decades have shown a decreasing prevalence of HF with reduced EF and an increasing incidence of HF with preserved EF, attributed to an aging population [3]. The elderly population in Türkiye increased by 49% in the last decade, with the proportion of elderly individuals in the total population rising to 9.5% in 2020^2^. Given these trends, it can be predicted that there will be a significant increase in the number of HF with preserved EF patients in the coming years. Consequently, NP measurement will be of great importance in supporting the diagnosis and differential diagnosis of these patients.

Although TTE is the indispensable imaging method for the diagnosis and classification of HF, other imaging methods are also occasionally needed. Chest X-ray and telecardiogram are traditionally employed during both diagnostic and follow-up phases, with a utilization rate of 93.7% in our country. The widespread availability of these examinations in almost all health institutions contributes to their high usage rates. Conversely, other imaging modalities, such as CMR, CTCA, and CAG, are used less frequently; investigations are primarily required for etiological evaluation. Since coronary heart disease is one of the most prevalent and treatable causes of HF [14, 15], a coronary angiography rate of 17.9% can be considered lower than expected. However, this rate is notably high compared to rates observed in other countries [16]. For instance, in EuroHeart survey, the frequency of CAG among hospitalized HF patients was 16% [16].

A recent study investigating temporal trends in the use of cardiac imaging for HF patients in Canada reported that the rate of CAG use was approximately 7%, remaining relatively stable from 2002 to 2016 [17]. Utilization of CTCA in Türkiye between 2016 and 2022 was 1.8%, which is higher compared to the 0.9% reported in 2016 in the aforementioned study (17). CTCA, being a non-invasive method to exclude coronary artery disease, has the potential to be used more frequently as an alternative to conventional CAG.

The use of CMR has increased worldwide over the years and is expected to continue increasing in the future [18]. Although CMR provides critical insights into the etiology of HF and myocardial tissue characteristics, difficulties in acquisition procedures, inadequate accessibility, and the low number of qualified personnel who can interpret CMR are the main problems limiting its widespread use [19]. In our country, the utilization of CMR is very low (0.3%) compared to rates in Western countries (e.g., 3% in Canada in 2016) [17]. This may be due to the aforementioned technical problems, as well as limited patient referrals by cardiologists. Enhancing the involvement of cardiology specialists in CMR imaging could potentially facilitate diagnosis..

Endomyocardial biopsy (EMB) is an invasive procedure utilized in a select group of patients, including those with myocarditis and those with a history of heart transplantation. The two most important factors limiting the use of EMB are its invasive procedure and the problems in evaluating the sample material. However, the increasing number of specialized and experienced pathologists in the field may facilitate more frequent clinical use of EMB.

This study should be interpreted considering certain limitations. The biochemical analysis results were not fully integrated into the database for the years 2016 and 2017, which may lead to inaccuracies in data for patients initially diagnosed with heart failure during those years. Additionally, some patients had both BNP and NT-proBNP measurements, potentially resulting in data duplication. Furthermore, temporal trends in the use of advanced imaging tests were not examined, which could reveal an increasing trend over the study period. Despite these limitations, our study provides the most comprehensive and up-to-date estimate of the utilization of diagnostic tools in our country.

## 5. Conclusion

Underdiagnosis of HF can result from several factors, including a lack of awareness of HF as a disease, underreporting of symptoms by patients, the nonspecific nature of HF symptoms or diagnostic uncertainty in the absence of a significant reduction in EF. Appropriate application of guideline directed diagnostic tools is crucial for effective HF management.. In Türkiye, the widespread use of TTE at the initial diagnosis and during follow-up period is essential for ensuring high-quality care for HF patients. Conversely, the utilization of detailed laboratory examinations and advanced imaging methods remains insufficient, potentially leading to issues in patient management.

Republic of Türkiye Social Security Institution provides comprehensive and equitable access to healthcare for all citizens, regardless of income level. Given that all laboratory and advanced imaging tests are fully reimbursed, the primary barrier to evidence-based patient care appears to be physicians’ attitudes. Enhancing physician awareness of routine diagnostic methods for HF may contribute to increasing the quality of patient care.

## Supplementary Information

**Supplementary Table S:** Distribution of provinces according to socioeconomic development ranking.

SES-1	SES-2	SES-3	SES-4	SES-5	SES-6

İstanbul	Denizli	Mersin	Amasya	Sinop	Adıyaman
Ankara	Sakarya	Trabzon	Hatay	Giresun	Ardahan
İzmir	Yalova	Adana	Nevşehir	Osmaniye	Diyarbakır
Kocaeli	Bolu	Zonguldak	Afyonkarahisar	Çankırı	Kars
Antalya	Konya	Uşak	Aksaray	Tokat	Iğdır
Bursa	Aydın	Gaziantep	Çorum	Niğde	Bingöl
Eskişehir	Isparta	Samsun	Artvin	Kahramanmaraş	Batman
Muğla	Kayseri	Burdur	Kastamonu	Tunceli	Şanlıurfa
Tekirdağ	Kırklareli	Kırıkkale	Erzincan	Ordu	Mardin
	Bilecik	Düzce	Bartın	Erzurum	Siirt
	Çanakkale	Karaman	Sivas	Kilis	Bitlis
	Edirne	Rize	Malatya	Yozgat	Van
	Karabük	Kütahya	Kırşehir	Gümüşhane	Hakkari
	Manisa		Elazığ	Bayburt	Muş
	Balıkesir				Ağrı
					Şırnak

SES, socioeconomical status.

## Figures and Tables

**Table 1:** Laboratory parameters of patients at the ‘initial’ diagnosis of heart failure.

Laboratory parameters	Male	Female	Total
	(n = 1,314,224)	(n = 1,407,927)	(n = 2,722,151)

***Tests routinely recommended***			

**BNP (pg/mL)**	948 (238–3273)	1000 (259–3722)	972 (248–3454)
	n = 89,325	n = 88,975	n = 178,300

**NT-proBNP (pg/mL)**	1417 (369–4359)	1332 (335–4379)	1374 (350–4366)
	n = 153,268	n = 152,641	n = 305,909

**Hemoglobin (g/dL)**	12.1 (9.3–14.1)	11.1 (8.8–12.7)	11.5 (9.0–13.4)
	n = 552,779	n = 605,613	n = 1,158,392

**Serum creatinine (mg/dL)**	0.99 (0.81–1.26)	0.83 (0.68–1.10)	0.93 (0.74–1.23)
	n = 493,888	n = 483,627	n = 977,515

**eGFR (mL/min/1.73 m** **^2^** **)**	71.39 (47.39–90.18)	64.93 (41.67–86.74)	68.13 (44.21–88.57)
	n = 493,888	n = 483,627	n = 977,515

**Serum sodium (mmol/L)**	137 (134–140)	138 (134–140)	137 (134–140)
	n = 442,362	n = 431,871	n = 874,233

**Serum potassium (mmol/L)**	4.1 (3.7–4.5)	4.1 (3.6–4.5)	4.1 (3.7–4.5)
	n = 465,264	n = 453,269	n = 918,533

**Fasting glucose (mg/dL)**	106.0 (91.0–137.0)	109.0 (93.0–141.9)	107.8 (92.0–139.0)
	n = 438,657	n = 435,326	n = 873,983

**HbA1c (%)**	6.5 (5.7–8.0)	6.5 (5.8–8.0)	6.5 (5.8–8.0)
	n = 55,702	n = 60,729	n = 116,431

**Triglyceride (mg/dL)**	116.0 (83.0–169.0)	123.0 (90.0–173.0)	120.0 (86.0–171.0)
	n = 225,407	n = 205,682	n = 431,089

**LDL cholesterol (mg/dL)**	99.0 (74.0–128.0)	106.0 (79.4–136.0)	102.3 (76.1–132.0)
	n = 261,641	n = 233,672	n = 495,313

**Ferritin (mL/ng)**	92.0 (38.0–211.4)	55.0 (23.4–132.4)	69 (24.0–210.1)
	n = 48,955	n = 62,555	n = 111,510

**TSH (mIU/L)**	1.29 (0.74–2.1)	1.45 (0.77–2.55)	1.37 (0.76–2.32)
	n = 209,497	n = 228,589	n = 438,086

***Tests not routinely recommended***			

**Albumin (g/dL)**	3.7 (3.1–4.3)	3.8 (3.2–4.3)	3.7 (3.1–4.3)
	n = 155,443	n = 159,153	n = 314,596

**Uric acid (mg/dL)**	6.1 (4.8–7.6)	5.9 (4.5–7.5)	5.9 (4.6–7.6)
	n = 179,215	n = 178,382	n = 357,597

BNP, brain natriuretic peptide; eGFR, estimated glomerular filtration rate; HbA1c, glycated hemoglobin; LDL, low density lipoprotein; NT-proBNP, N-terminal pro-BNP; TSH, thyroid-stimulating hormone.

* Median values (25th–75th percentile).

**Table 2:** Natriuretic peptide levels in patients with heart failure according to age groups.

	Age group (years)			

	0–19	20–49	50–74	≥75
	(n = 23,443)	(n = 201,773)	(n = 1,524,175)	(n = 972,760)

**BNP (pg/mL)**	1114 (124–5000)	367 (72–1803)	797 (201–2709)	1475 (432–4983)
	n = 2366 (10.0%)	n = 13,116 (6.5%)	n = 102,841 (6.7%)	n = 59,977 (6.2%)

**NT-proBNP (pg/mL)**	1387 (180–5336)	533 (102–2505)	1078.0 (278–3429)	2150 (636–6184)
	n = 3879 (16.5%)	n = 23,633 (11.7%)	n = 175,877 (11.5%)	n = 102,520 (10.5%)

BNP, brain natriuretic peptide; NT-proBNP, N-terminal pro-BNP.

* Median values (25th–75th percentile).

**Table 3:** Frequency of natriuretic peptide measurements according to comorbidities.

	NPs n, (%)
**DM (n = 1,230,323)**	231,888 (18.8)
**Anemia (n = 1,105,450)**	198,886 (18.0)
**Atrial fibrillation (n = 1,010,148)**	207,463 (20.5)
**AMI (n = 692,154)**	112,715 (19.0)
**Obesity (n = 93,925)**	20,130 (21.4)
**COPD (n = 1,185,821)**	224,498 (18.9)
**CKD (n = 481,776)**	111,111 (23.1)

AMI, acute myocardial infarction; COPD, chronic obstructive pulmonary disease; CKD, chronic kidney disease; DM, diabetes mellitus; HT, hypertension; eGFR, estimated glomerular filtration rate; NPs, natriuretic peptides.

**Table 4:** Natriuretic peptide levels according to estimated glomerular filtration rate in patients with heart failure.

	eGFR (mL/min/1.73 m^2^)			

	<15	15–29	30–59	≥60
	(n = 22,708)	(n = 47,252)	(n = 239,017)	(n = 650,553)

**BNP (pg/mL)**	5000	4414	1727	777
	(1231–5000)	(975–5000)	(482–5000)	(197–2600)

**NT-proBNP (pg/mL)**	14524	7669	2606	1063
	(3259–35000)	(2454–22000)	(745–7697)	(277–3133)

BNP, brain natriuretic peptide; eGFR, estimated glomerular filtration rate; NT-proBNP, N-terminal pro-BNP.

* Median values (25th–75th percentile).

**Table 5:** Imaging modalities and invasive diagnostic tests used in the diagnosis and during follow-up of patients with heart failure.

Diagnostic modality	Male (n = 1,314,224)	Female (n = 1,407,927)	Total (n = 2,722,151)	EuroHeart Failure Survey 2003*
***Routinely recommended***				
**ECG n, (%)**	1,314,224 (100.0)	1,407,927 (100.0)	2,722,151 (100.0)	(94)
**PA chest radiography n, (%)**	1,230,380 (93.6)	1,321,152 (93.8)	2,551,532 (93.7)	(79)
**TTE n, (%)**	1,314,224 (100.0)	1,407,927 (100.0)	2,722,151 (100.0)	(34)
***Not routinely recommended***				
**Conventional CAG n, (%)**	294,571 (22.4)	193,248 (13.7)	487,819 (17.9)	(16)
**CCTA n, (%)**	23,907 (1.8)	25,603 (1.8)	49.510 (1.8)	Not applicable
**CMR n, (%)**	4759 (0.4)	3079 (0.2)	7.828 (0.3)	Not applicable
**RHC n, (%)**	3892 (0.3)	4471 (0.3)	8.363 (0.3)	Not applicable
**EMB n, (%)**	247 (0.0)	107 (0.0)	354 (0.0)	Not applicable

TTE, transthoracic echocardiography; ECG, electrocardiography; PA, posteroanterior; CAG, coronary angiography; CCTA, coronary computed tomographic angiography; CMR, cardiac magnetic resonance; EMG, endomyocardial biopsy.

* Percentage of diagnostic test usage in the “EuroHeart Failure survey programme — a survey on the quality of care among patients with heart failure in Europe” was shown (16).

**Table 6:** Number of natriuretic peptides measurements and utility of imaging modalities at the diagnosis and during the follow-up of the patients with heart failure according to socioeconomic development index (SEDI) groups of provinces in Türkiye.

SEDI groups	NP	CXR	CTCA	CMR	RHC	EMB	CAG
	n, (%)	n, (%)	n, (%)	n, (%)	n, (%)	n, (%)	n, (%)

**SES-1**	242.397	1.041.950	16.360	5.203	3558	216	176.101
	(21.6)	(93.0)	(1.5)	(0.5)	(0.3)	(0.0)	(15.7)

**SES-2**	50.230	444.367	3.916	986	779	25	84.927
	(10.6)	(94.0)	(1.8)	(0.0)	(0.2)	(0.0)	(17.9)

**SES-3**	54.997	401.855	6.645	717	914	55	76.309
	(12.9)	(94.3)	(1.6)	(0.1)	(0.2)	(0.0)	(17.9)

**SES-4**	43.701	248.417	8.646	327	991	18	50.782
	(16.5)	(93.7)	(3.3)	(0.1)	(0.4)	(0.0)	(19.2)

**SES-5**	32.784	226.012	7.299	239	528	19	47.390
	(13.8)	(95.1)	(3.1)	(0.1)	(0.2)	(0.0)	(19.9)

**SES-6**	19.226	188.931	6.644	366	1693	21	52.310
	(9.6)	(94.5)	(3.3)	(0.2)	(0.8)	(0.0)	(26.2)

CXR, chest X-ray; CAG, coronary angiography; CTCA, computed tomography coronary angiography; CMR, cardiac magnetic resonance; EMG, endomyocardial biopsy; NP, natriuretic peptide; RHC, right heart catheterization; SEDI, socioeconomic development index; SES, socioeconomic status.

## Footnotes

1 Ankara Kalkınma Ajansları Genel Müdürlüğü Yayını (2019). İllerin ve bölgelerin sosyo-ekonomik gelişmişlik sıralaması araştırması [online]. Website https://www.bebka.org.tr/admin/datas/sayfas/89/sege-2017_1581687211.pdf [accessed 1 December 2019].

2 Türkiye İstatistik Kurumu (2021). İstatistiklerle yaşlılar, 2020 [online]. Website https://data.tuik.gov.tr/Bulten/Index?p=Istatistiklerle-Yaslilar-2020-37227 [accessed 18 March 2021].
